# miR‐124‐3p availability is antagonized by LncRNA‐MALAT1 for Slug‐induced tumor metastasis in hepatocellular carcinoma

**DOI:** 10.1002/cam4.2482

**Published:** 2019-08-29

**Authors:** Rong‐Jun Cui, Jia‐Lin Fan, Yu‐Cui Lin, Yu‐Jia Pan, Chi Liu, Jia‐Hui Wan, Wei Wang, Zheng‐Yuan Jiang, Xiu‐Lan Zheng, Jie‐Bing Tang, Xiao‐Guang Yu

**Affiliations:** ^1^ Department of Biochemistry and Molecular Biology Harbin Medical University Harbin Heilongjiang China; ^2^ Department of Biochemistry and Molecular Biology Mudanjiang Medical University Mudanjiang Heilongjiang China; ^3^ The Second People's Hospital of Lishui Lishui Zhejiang China; ^4^ Department of Ultrasonography Harbin Medical University Cancer Hospital Harbin Heilongjiang China; ^5^ Department of Gastrointestinal Medical Oncology Harbin Medical University Cancer Hospital Harbin Heilongjiang China

**Keywords:** hepatocellular carcinoma, metastasis‐associated lung adenocarcinoma transcript 1, miR‐124‐3p, tumor metastasis

## Abstract

**Background:**

As an oncogene, long noncoding RNA metastasis‐associated lung adenocarcinoma transcript 1 (MALAT1) can promote tumor metastasis. Hyperexpression of MALAT1 has been observed in many malignant tumors, including hepatocellular carcinoma (HCC). However, the role and mechanism of MALAT1 in HCC remain unclear.

**Methods:**

Thirty human HCC and paracancerous tissue specimens were collected, and the human hepatoma cell lines Huh7 and HepG2 were cultured according to standard methods. MALAT1 and Snail family zinc finger (Slug) expression were measured by real‐time PCR, immunohistochemistry, and western blotting. Luciferase reporter assay and RNA immunoprecipitation (RIP) assay verified the direct interaction between miR‐124‐3p and Slug(SNAI2) or MALAT1. Wound healing and transwell assays were performed to examine invasion and migration, and a subcutaneous tumor model was established to measure tumor progression in vivo.

**Results:**

MALAT1 expression was upregulated in HCC tissues and positively correlated with Slug expression. MALAT1 and miR‐124‐3p bind directly and reversibly to each other. MALAT1 silencing inhibited cell migration and invasion. miR‐124‐3p inhibited HCC metastasis by targeting Slug.

**Conclusions:**

MALAT1 regulates Slug through miR‐124‐3p, affecting HCC cell metastasis. Thus, the MALAT1/miR‐124‐3p/Slug axis plays an important role in HCC.

## Graphical abstract

Metastasis‐associated lung adenocarcinoma transcript 1 (MALAT1) regulates Slug through miR‐124‐3p, affecting cell migration and cell invasion. Thus, the MALAT1/miR‐124‐3p/Slug axis plays an important role in hepatocellular carcinoma.

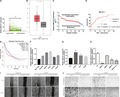

## 1 INTRODUCTION

Liver cancer is the most common solid cancer worldwide, especially in China.^1^ This disease is a major health problem, with more than 841 000 new cases and 782 000 deaths annually worldwide.^2^ Hepatocellular carcinoma (HCC) is the most common primary liver cancer, accounting for approximately 90% of cases.^3^ Effective treatment is lacking, and patients mostly die from intrahepatic or extrahepatic metastasis. Metastasis is often accompanied by the development of new tumors.^4^ Therefore, studies of the metastatic mechanisms of HCC are helpful for elucidating HCC metastasis factors and identifying new therapeutic targets.

For years, studies have focused on protein coding genes because these genes are widely involved in the mechanisms of cancer initiation and progression. Since the success of the Human Genome Sequencing Project, ncRNAs have attracted researchers’ interest. Long noncoding RNAs (lncRNAs) are a group of RNAs that have transcript lengths greater than 200 nts.^5^ Recent studies have shown that lncRNAs participate in many important physiological activities, such as genomic imprinting, chromatin modification, transcriptional activation, posttranscriptional regulation, and protein functional regulation, and these findings have progressively underscored the importance of cancer research.^6^, ^7^

Metastasis‐associated lung adenocarcinoma transcript 1 (MALAT1) is a long intergenic nonprotein coding RNA that is over 8000 nts in length and located on chromosome 11q13. MALAT1 sequences are highly conserved among species, which predicts their potentially important biological functions.^8^ MALAT1 expression is upregulated in many types of tumors, and MALAT1 exhibits effects on proliferation, migration, invasion, and apoptosis.^9^, ^10^ Abnormal MALAT1 expression has been observed in almost every organ of the digestive system.^11^, ^12^, ^13^, ^14^

We show that the lncRNA MALAT1 is upregulated in HCC tissues and correlated with poor prognosis in this study. The experimental results show that MALAT1 promotes HCC cell migration and invasion as a competing endogenous RNA (ceRNA) for miR‐124‐3p, thereby blocking its association with its target mRNA, namely, Snail family zinc finger (Slug). Collectively, these results show that MALAT1 is an oncogenic HCC tumorigenesis gene and may be an effective candidate gene for HCC diagnosis and therapy.

## 2 MATERIALS AND METHODS

### 2.1 Patients and specimens and ethics statement

The tumor tissue cDNA arrays for human HCC (cDNA‐HLivH60PG01) and the tumor tissue arrays for human HCC (HLivH030PG03) were provided by Shanghai Outdo Biotech (Shanghai, China), and there were 30 samples in each tissue microarray, including 15 cases of liver cancer tissues and the pair‐matched adjacent tissues. The Ethics Committee of Taizhou Hospital of Zhejiang Province authorized all experiments on patient tissues in this study. The Ethics Committee of Harbin Medical University authorized all animal experiments in this study.

### 2.2 Immunohistochemistry

Tissue microarrays are routinely subjected to immunohistochemistry (IHC). The immunohistochemical score was determined based on the percentage of positively stained cells and the staining density. The results were scored separately by three pathologists who were unfamiliar with the patient conditions.

### 2.3 Cell culture

The cell lines, including HepG2, HuH7, and HEK293T, were purchased separately from the Cell Bank of the Chinese Academy of Science. The cell lines were cultured in RPMI‐1640 medium containing 10% FBS, 100 U/mL penicillin, and streptomycin and were maintained in a humidified incubator containing 5% CO_2_ at 37°C.

### 2.4 RNA extraction, RT and qPCR

RNA (including miRNA) was extracted using a TRIzol kit (Life Technologies) according to the product instructions. The extracted RNA was reverse transcribed into cDNA using a PrimeScript RT kit (TaKaRa Bio), and genomic DNA was removed according to the product instructions. cDNA template amplification was performed using SYBR Select Master Mix (Life Technologies). The expression of GAPDH was used as an internal reference. The primer sequences used were as follows: GAPDH‐forward, 5′‐ACCCAGAAGACTGTGGATGG‐3′ and GAPDH‐reverse, 5′‐CAGTGAGCTTCCCGTTCAG‐3′; MALAT1‐forward, 5′‐CTTCCCTAGGGGATTTCAGG‐3′ and MALAT1‐reverse, 5′‐GCCCACAGGAACAAGTCCTA‐3′; Slug‐forward, 5′‐TGCGATGCCCAGTCTAGAAA‐3′ and Slug‐reverse, 5′‐AAAAGGCTTCTCCCCCGTGT‐3′. Reactions for real‐time PCR analysis were performed on an ABI 7500 system (Applied Biosystems). The relative fold change in mRNA expression was calculated according to the 2^−ΔΔCt^ method.

### 2.5 Cell transfection

siRNAs, to specifically knock down MALAT1 were produced by Suzhou GenePharma Co., Ltd. The siRNA sequences targeting MALAT1 were as follows: sense‐1, 5′‐GCAAAUGAAAGCUACCAAUTT‐3′; antisense‐1, 5′‐AUUGGUAGCUUCAUUUGCTT‐3′; sense‐2, 5′‐GCCGAAAUAAAUGAGAGAUTT‐3′; antisense‐2, 5′‐AUCUCUCAUUUAUUUCGGCTT‐3′; sense‐3, 5′‐GCUGUGGAGUUCUUAAAUATT‐3′; and antisense‐3, 5′‐UAUUUAAGAACUCCAGCTT‐3′. The negative control (NC) siRNA sequences were as follows: sense, 5′‐UUCUCCGAACGUGUCACGUTT‐3′ and antisense, 5′‐ACGUGACACGUUCGGAGAATT‐3′.

Transfections were performed using LipofectamineTM 2000 (Invitrogen) according to the manufacturer's instructions. After siRNA plasmid transfection at a working concentration of 100 nM, the cells were incubated for 48 hours. miR‐124‐3p mimics and inhibitors (Guangzhou RiboBio Co., Ltd.) were also transfected into HepG2 and Huh7 cells using LipofectamineTM 2000 as a transfection reagent.

### 2.6 Dual luciferase reporter assay

This assay was performed as previously described.^15^ Human HEK293T cells were cotransfected with the appropriate empty plasmid, MALAT1‐wt or MALAT1‐mut. The cells were also cotransfected with miR‐124‐3p mimics or miRNA NC using the same methods. Similarly, Slug‐wt and Slug‐mut were transfected into HEK293T cells. Renilla fluorescence activity was used as an internal reference, and the RLU ratio was calculated. On the basis of software prediction, the binding sites of miR‐124‐3p to MALAT1 and Slug were detected and verified.

### 2.7 RNA immunoprecipitation

This experiment was performed using an RNA‐Binding Protein Immunoprecipitation Kit (Millipore) in strict accordance with the product manual. Protein A magnetic beads were ligated to the antibody at 4°C and then incubated with HepG2 and Huh7 cell lysates. After 5 hours, the beads were washed to remove residual protein and incubated with proteinase K at 55°C for 30 minutes. Then, RNA was extracted and subjected to real‐time PCR analysis.

### 2.8 Xenograft mouse model

The animal experiments in this paper were all approved by the Ethics Committee of Harbin Medical University. HepG2 cells stably expressing control shRNA or shRNA‐MALAT1 were prepared. Approximately 1 × 10^7^ of the above HepG2 cells were injected subcutaneously into the armpits of male BALB/c nude mice. Four weeks later, the mice were sacrificed by spinal cord dislocation. The tumors were removed, and routine H&E staining and immunohistochemical staining were performed.

### 2.9 Statistical analysis

The data in this paper are expressed as the mean ± standard deviation. Student's *t* test was used to measure significant differences. The Pearson correlation coefficient was used to analyze the correlations. *P* < .05 was considered to represent a statistically significant difference.

## 3 RESULTS

### 3.1 Increased MALAT1 expression levels in HCC tissues are associated with malignant features and low disease‐free survival

**Figure 1:**
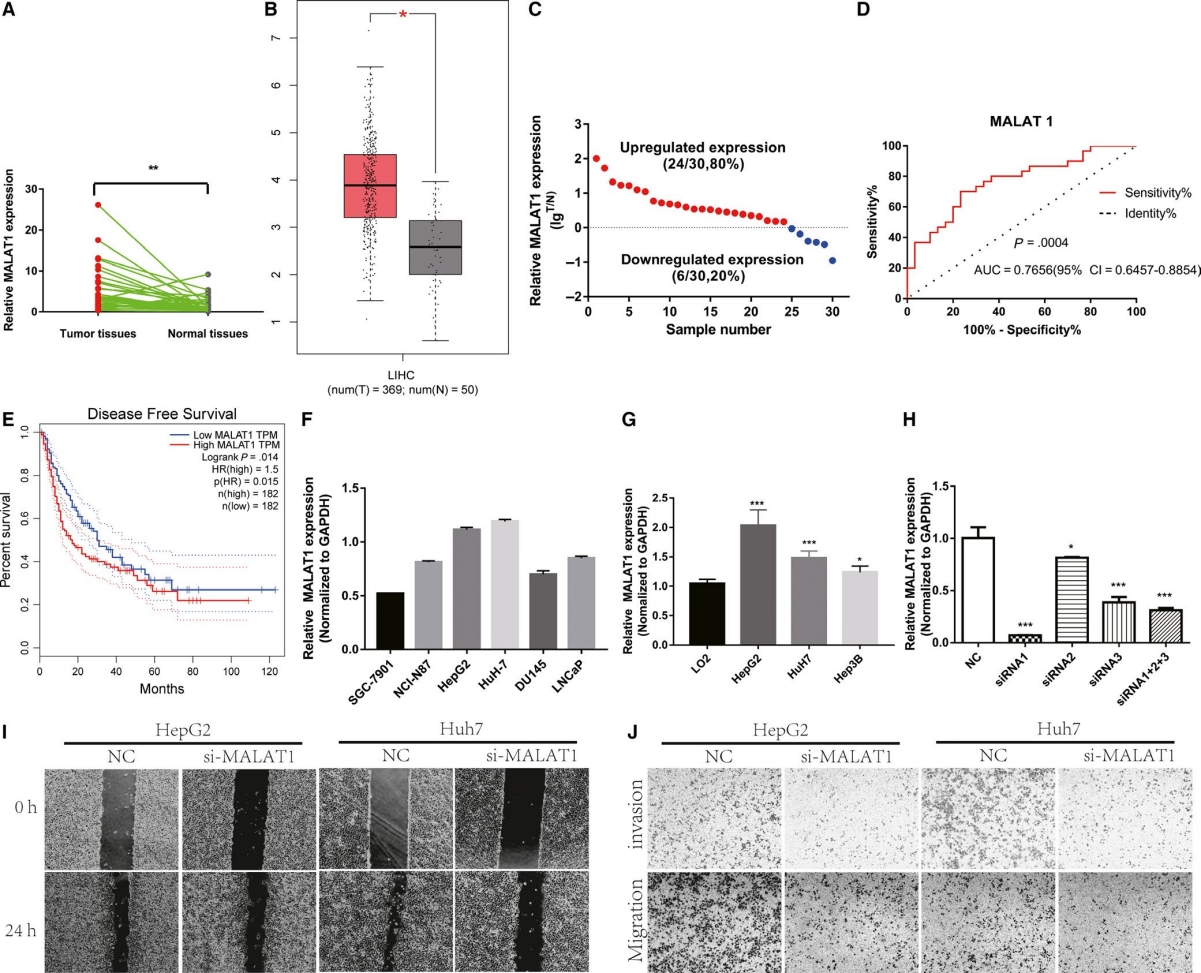
MALAT1 expression and its significance in HCC tissue and cell lines. (A) MALAT1 expression in HCC tissue and precancerous tissue (**P < .01). (B) Relative MALAT1 expression in HCC tissue from TCGA database (*P < .05). (C) Upregulated and downregulated expression of MALAT1 in HCC tumor tissue and normal tissue (T: tumor; N: normal). (D) ROC curve of MALAT1 expression in HCC tissues and precancerous tissues. (E) Kaplan‐Meier disease‐free survival curves according to MALAT1 expression level from TCGA database. Overall survival of the high‐MALAT1 group (n = 182: MALAT1 expression ratio ≥ median ratio) was significantly higher than that of the low‐MALAT1 group (n = 182; MALAT1 expression ratio ≤ median ratio; *P* = .015, log rank test). (F) MALAT1 expression in stomach carcinoma, prostate cancer cell lines and HCC cell lines. (G) MALAT1 expression in HCC cell lines and the normal liver LO2 cell line (*P < .05; ***P < .001). (H) HepG2 cells transfected with si‐MALAT1 were analyzed by real‐time PCR for MALAT1 expression (*P < .05; ***P < .001). The effect of MALAT1 on HCC cell migration and invasion ability was assessed with the scratch wound (I) and transwell chamber assays (J). HCC, hepatocellular carcinoma; MALAT1, metastasis‐associated lung adenocarcinoma transcript 1

The expression levels of MALAT1 were higher in HCC tissues than in adjacent normal tissues (Figure 1A). The same results were obtained with TCGA data (Figure 1B).^16^ As shown in Figure 1C, 24 HCC tissues (80%) had upregulated (lgT/N > 0) MALAT1 expression, whereas only six HCC tissues had downregulated (lgT/N < 0) MALAT1 expression (*P* < .001). ROC curves for MALAT1 expression were established to distinguish HCC tissue from normal liver tissue (Figure 1D). As shown, we identified a lncRNA signature for MALAT1, which could act as a potential independent biomarker for prognostic HCC predictions. Thirty patients with HCC were divided into two groups based on MALAT1 expression values. Those with a MALAT1 expression value higher than or equal to the median (3.09) were included in the high MALAT1 group (n = 15); those with a lower MALAT1 expression value than the median were included in the low MALAT1 group (n = 15). There was no correlation between MALAT1 upregulation and sex, age, pathological grade, or lesion location. However, high MALAT1 expression was positively correlated with tumor size, MVI, and poor differentiation status (Table 1). We also found that high MALAT1 expression was negatively correlated with disease‐free survival (DFS) in HCC patients based on TCGA data and negatively correlated with DFS (Figure 1E).

**Table 1:** Correlation between MALAT1 expression and clinical features in 30 patients with HCC

Clinical characteristics	Case number	Low MALAT1 expression	High MALAT1 expression	*P*‐value
Sex				.142
Male	25	11	14	
Female	5	4	1	
Age				.837
≤60 y	16	8	8	
>60 y	13	6	7	
Tumor size				.013*
≤5 cm	6	4	2	
>5 cm	24	4	20	
MVI				.038*
No	13	10	3	
Yes	16	5	11	
Differentiation status				.017*
Poor	11	3	8	
High/moderate	19	15	4	
TNM stage				.46
I‐II	17	10	7	
III‐IV	13	5	8	
Pathological grade				.159
II	22	9	13	
II‐III/III	7	5	2	
Lesion location				.178
Left lobe of the liver	7	5	2	
Right lobe of the liver	17	7	10	

Abbreviation: MALAT1, metastasis‐associated lung adenocarcinoma transcript 1.

* *P* < .05.

### 3.2 MALAT1 promotes HCC cell metastasis

Expression levels of MALAT1 in six common solid malignant cell lines (stomach carcinoma: SGC‐7901 and NCL‐N87; hepatocarcinoma: HepG2 and Huh‐7; prostate cancer: DU145 and LNCaP) were detected. As shown in Figure 1F, after normalization to GAPDH expression, MALAT1 expression levels were highest in HepG2 and Huh‐7 cells (*P* < .001). We then examined MALAT1 expression in human liver cell lines and HCC cell lines; the MALAT1 levels were significantly increased in the HCC cell lines (Figure 1G). Next, we altered the expression of MALAT1 in HepG2 and Huh‐7 cells to investigate the biological role of MALAT1 in liver cancer. Three separate MALAT1 siRNAs were synthesized and transfected into HepG2 cell lines, either separately or in combination. MALAT1 expression was significantly lower in si‐MALAT1 transfected cells than in control cells (*P* < .001). siRNA1 decreased MALAT1 expression to the greatest extent, so siRNA1 was used for subsequent experiments (Figure 1H). Transwell assays and scratch wound assays showed significantly reduced invasion and migration abilities of cells with downregulated MALAT1 expression compared to those of the control cells (Figure 1I,J). These results indicate that MALAT1 promotes the migration and invasion of HCC cells.

### 3.3 The promotion of cell invasion and metastasis by MALAT1 depends on miR‐124‐3p in HCC cells

**Figure 2:**
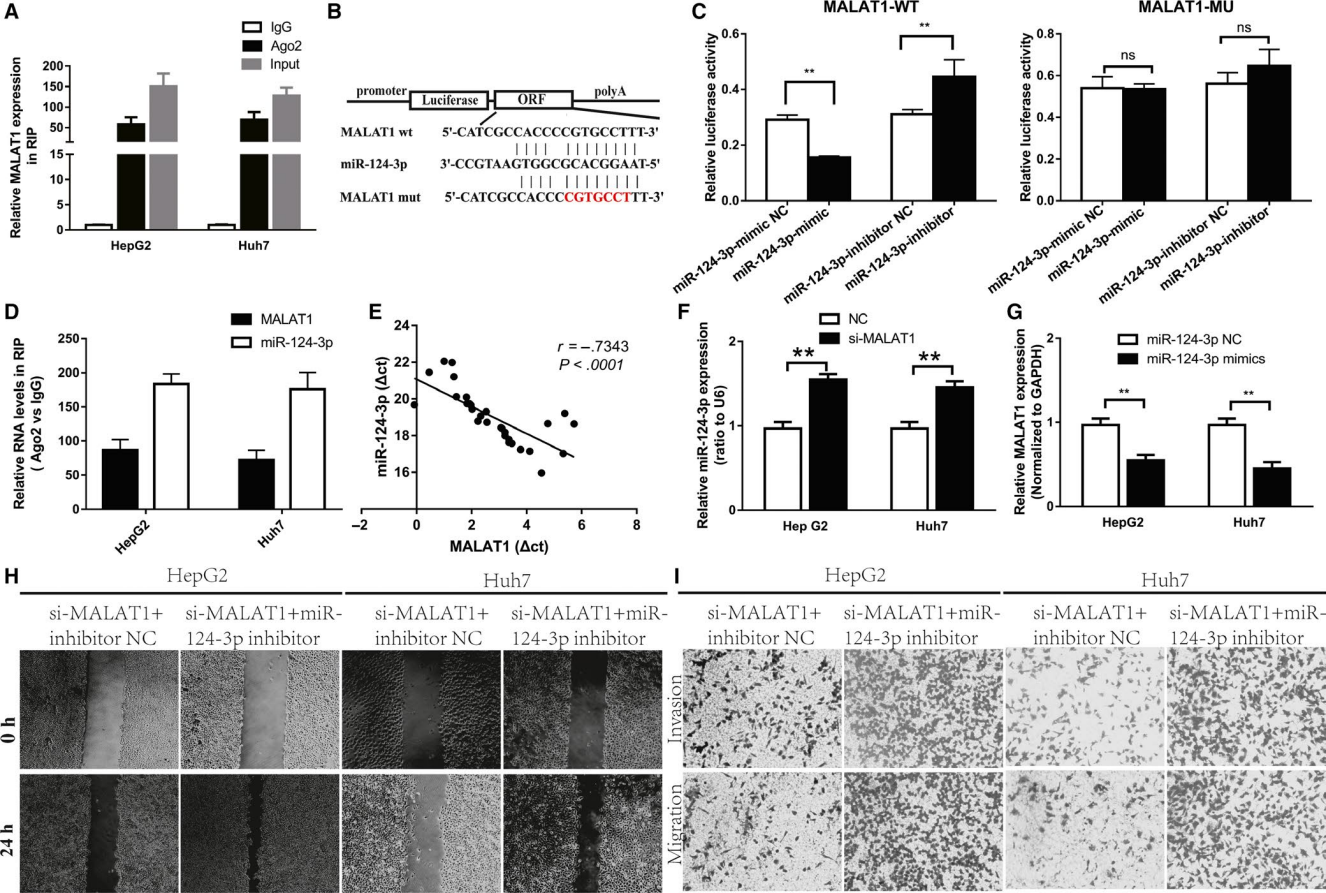
miR‐124‐3p is a target of MALAT1 in HCC. (A) RIP experiments were performed in HepG2 and Huh7 cells, and the coprecipitated RNA was subjected to real‐time PCR for MALAT1. (B) Bioinformatic analysis showed that miR‐124‐3p directly targeted the MALAT1‐WT (wild‐type) sequence. MALAT1‐Mut (mutant) contained mutations in the binding sites within the MALAT1 sequence. (C) Dual luciferase reporter assays showed that miR‐124‐3p negatively regulated the luciferase activity of MALAT1‐WT but not that of MALAT1‐Mut (**P < .01). (D) RNA levels in immunoprecipitates are presented as the fold enrichment in Ago2 immunoprecipitates relative to that in IgG immunoprecipitates. (E) The relative expression of miR‐124‐3p in HCC tissues compared with that in normal tissues was analyzed using a cDNA array of 30 paired HCC tissues. (F) Real‐time PCR analysis of miR‐124‐3p expression in HepG2 and Huh7 cells transfected with control siRNA or MALAT1 siRNA (**P < .01). (G) Real‐time PCR analysis of MALAT1 expression in HepG2 and Huh7 cells transfected with control siRNA or the miR‐124‐3p mimic (**P < .01). (H) Wound healing migration and transwell assays (I) were evaluated in HCC cells transfected with si‐MALAT1 + inhibitor NC and si‐MALAT1 + miR‐124‐3p inhibitor. HCC, hepatocellular carcinoma; MALAT1, metastasis‐associated lung adenocarcinoma transcript 1; NC, negative control; RIP, RNA immunoprecipitation

Many previous studies have shown that lncRNAs can function as ceRNAs in carcinogenesis. The ceRNA theory suggests that certain lncRNAs can bind to specific miRNAs by partial hybridization, rendering the miRNA to be unable to bind to its target gene, thereby causing the target mRNA to lose the inhibition of the miRNA.^17^, ^18^ Therefore, we considered whether MALAT1 had the same effect in HCC cell lines. As expected, RNA immunoprecipitation (RIP) testing of HCC cell extracts confirmed our hypothesis. MALAT1 and Ago2 can bind directly (Figure 2A). Ago2 is involved in miRNA‐mediated mRNA suppression and is a component of the RNA‐induced silencing complex. This means that MALAT1 can act as a ceRNA for specific miRNAs. To further validate this hypothesis, we used the online bioinformatics database Starbase 2.0^19^ and observed that the MALAT1 sequence contains potential miR‐124‐3p‐binding sites (Figure 2B). Next, luciferase reporter plasmids containing a mutant MALAT1 sequence (the site of the mutation was the miR‐124‐3p binding site in the MALAT1 molecular sequence predicted by bioinformatics) or the wild‐type MALAT1 sequence were constructed. The luciferase activity of MALAT1‐WT was shown to be decreased in the dual luciferase reporter assay results, and there was no change in the luciferase activity of MALAT1‐MUT in HEK293 cells (Figure 2C). Additionally, RNA‐binding protein immunoprecipitation experiments showed that miR‐124‐3p and MALAT1 were significantly enriched in the immunoprecipitates of Ago2 relative to those of the control IgG immunoprecipitates (Figure 2D). Notably, real‐time PCR analysis of 30 HCC tissues demonstrated a significant negative correlation between miR‐124‐3p and MALAT1 levels (Figure 2E). Real‐time PCR showed that MALAT1 knockdown caused a significant increase in miR‐124‐3p expression levels (Figure 2F), and MALAT1 expression was decreased in cells transfected with miR‐124‐3p mimics. (Figure 2G). These results indicate that MALAT1 and miR‐124‐3p have a negative regulatory relationship in HCC cells. Functional miR‐124‐3p experiments were performed using HepG2 and Huh7 cells. The results showed that migration and invasion abilities were upregulated in the si‐MALAT1 + miR‐124‐3p inhibitor groups (Figure 2H,I). In HCC cells, these properties were attenuated by si‐MALAT1. As expected, these data indicate that MALAT1’s function depends partially on miR‐124‐3p.

### 3.4 miR‐124‐3p inhibits HCC cell metastasis

**Figure 3:**
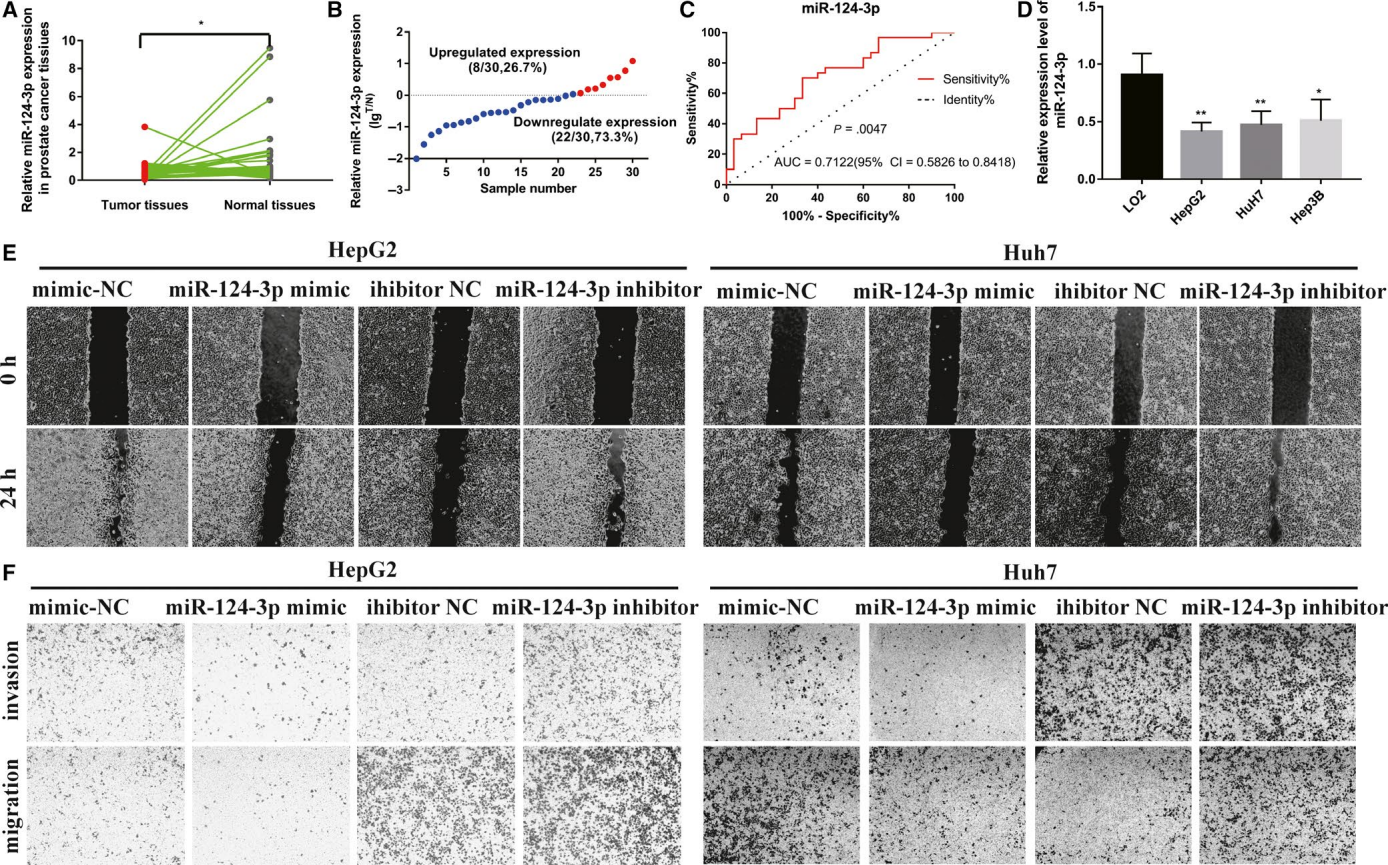
miR‐124‐3p serves as a tumor suppressor in HCC. (A) miR‐124‐3p expression in HCC tissue and precancerous tissue (*P < .05). (B) Upregulated and downregulated expression of miR‐124‐3p in HCC tumor tissue and normal tissue (T: tumor; N: normal). (C) ROC curve of miR‐124‐3p expression in HCC tissue and precancerous tissue. (D) Expression levels of miR‐124‐3p were evaluated by real‐time PCR in normal liver and HCC cell lines (*P < .05, **P < .01). Effect of miR‐124‐3p on HCC cell migration and invasion ability was assessed with scratch wound (E) and transwell chamber assays (F). HCC, hepatocellular carcinoma

The miR‐124‐3p level was higher in HCC tissues than in adjacent normal tissues (Figure 3A). As shown in Figure 3B, only six HCC tissues (26.7%) showed upregulated (lgT/N > 0) miR‐124‐3p expression, whereas 22 HCC tissues showed downregulated (lgT/N < 0) miR‐124‐3p expression (*P* < .001). ROC curves for miR‐124‐3p were generated to distinguish HCC tissue from normal liver tissue (Figure 3C). miR‐124‐3p levels were significantly reduced in HCC cell lines (Figure 3D). We evaluated HCC invasion and migration in HepG2 and Huh7 cells using scratch wound and transwell chamber assays. As shown in Figure 3E,F, invasion and migration were significantly decreased when miR‐124‐3p was upregulated in HepG2 and Huh7 cells. However, downregulation of miR‐124‐3p increased the migration and invasion of HepG2 and Huh7 cells. Thus, we demonstrated that miR‐124‐3p could act as a potential independent biomarker for prognostic HCC progression predictions.

### 3.5 Slug is a direct downstream target of miR‐124‐3p in HCC

**Figure 4:**
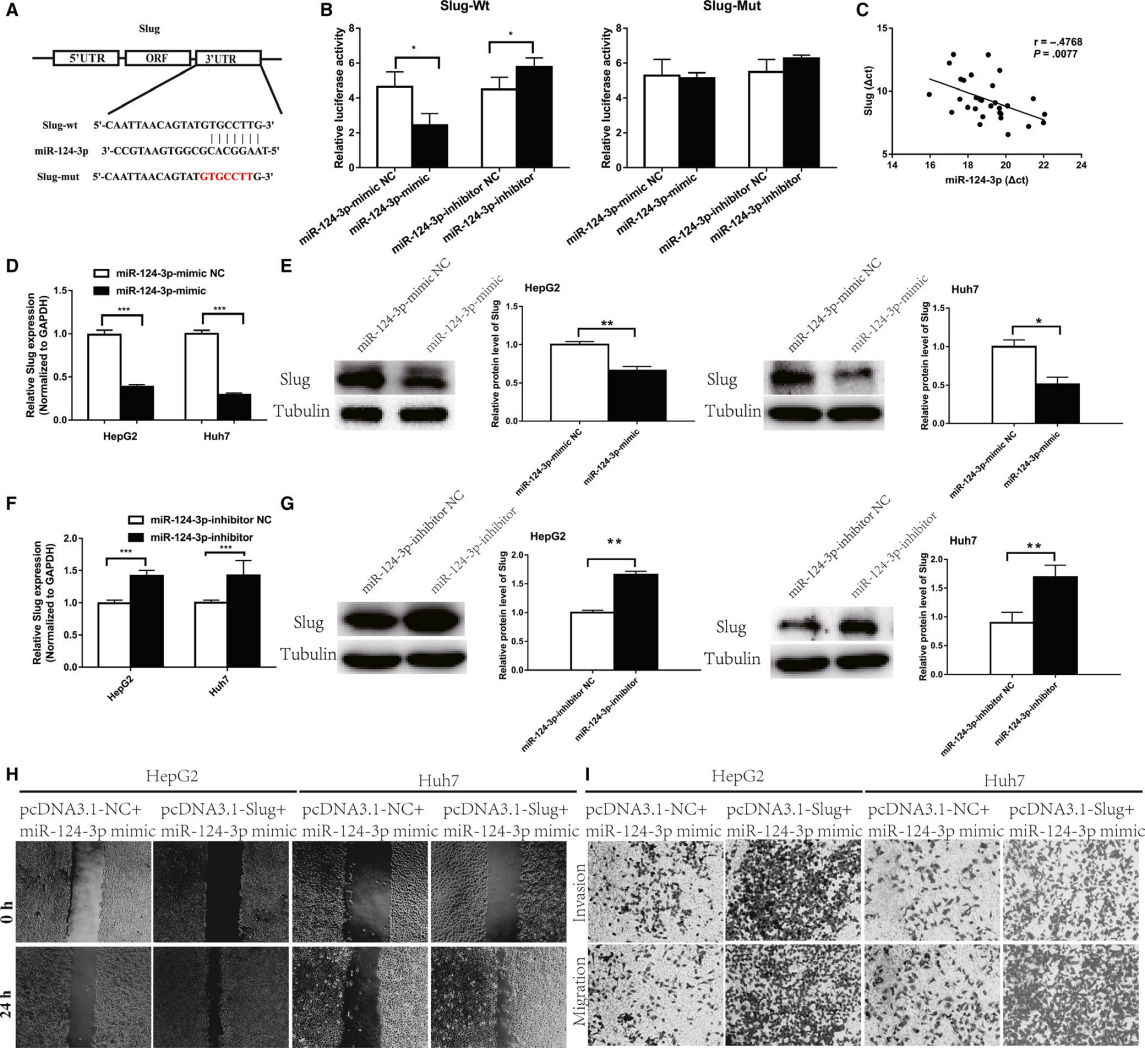
miR‐124‐3p directly targets Slug. (A) Sequence complementarity between Slug and miR‐124‐3p. Luciferase reporter constructs containing the Slug or mutated Slug sequence. (B) Slug‐Wt and Slug‐Mut were each cotransfected into HEK293 cells with miR‐124‐3p mimics or inhibitors. Luciferase activity was determined 48 h after transfection. (*P < .05) (C) Pearson's correlation between miR‐124‐3p and Slug in HCC and normal tissues. (D‐G) Mimics or inhibitors were transfected into HepG2 and Huh‐7 cells, and Slug expression was detected by real‐time PCR and Western blot. (*P < .05, **P < .01, ***P < .001). (H) Wound healing migration and transwell assays (I) were evaluated in HCC cells transfected with the pcDNA3.1‐NC + miR‐124‐3p and pcDNA3.1‐Slug + miR‐124‐3p mimics. HCC, hepatocellular carcinoma; NC, negative control

As shown in an online database, Slug may be an alternative downstream target for miR‐124‐3p (Figure 4A).^19^ We constructed a mutated Slug3′‐UTR, and luciferase activity was demonstrated using a dual luciferase reporter assay. The results showed that the luciferase activity was decreased by only wild‐type Slug and not by mutant Slug (*P* < .001, Figure 4B). Notably, real‐time PCR analysis of 30 HCC tissues revealed a significant negative correlation between miR‐124‐3p and Slug levels (Figure 4C). In vitro, we demonstrated that the upregulation of miR‐124‐3p inhibited Slug mRNA and protein expression in HCC cells (Figure 4D‐G). As previously stated, miR‐124‐3p reduces HCC cell invasion and metastasis. However, does miR‐124‐3p's function depend on its target gene, Slug? We overexpressed Slug in HCC cells overexpressing miR‐124‐3p and found that Slug restored the altered invasion and metastatic potential caused by the miR‐124‐3p mimic. This suggests that the biological function of miR‐124‐3p is partially dependent on its target gene, Slug.

### 3.6 Increased Slug expression levels in HCC tissues are associated with poor prognosis

**Figure 5:**
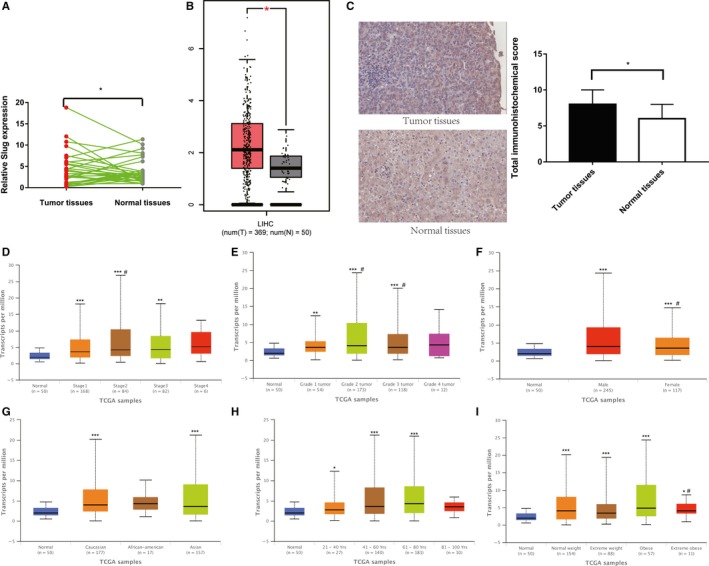
Relative Slug expression and its clinical significance in liver cancer tissues. (A) Slug expression in HCC tissues and precancerous tissues (*P < .05). (B) Relative Slug expression in HCC tissues from TCGA database. (*P < .05). (C) IHC staining of Slug in HCC tissues (*P < .05). (D) Slug expression in HCC based on individual cancer stages. Compared with normal, **P < .01 and ***P < .001; compared with Stage 1, ^#^ *P* < .05. (E) Slug expression in HCC based on grade. Compared with normal, **P < .01 and ***P < .001; compared with Grade 4, ^#^ *P* < .05. (F) Slug expression in HCC based on patient's sex. Compared with normal, ***P < .001; compared with Male, ^#^ *P* < .05. (G) Slug expression in HCC based on the patient's race. Compared with normal, ***P < .001. (H) Slug expression in HCC based on the patient's age. Compared with normal, *P < .05 and ***P < .001. (I) Slug expression in HCC based on the patient's weight. Compared with normal, *P < .05 and ***P < .001; compared with extreme weight loss, ^#^ *P* < .05. HCC, hepatocellular carcinoma; IHC, immunohistochemistry

The expression levels of Slug in HCC and relative matched adjacent normal tissues (n = 30) were measured by real‐time PCR analysis to verify the role of Slug in HCC. The results indicate that the expression levels of Slug were higher in HCC tissues than in matched adjacent normal tissues. (Figure 5A). The same tendency was observed in the TCGA database (Figure 5B).^6^ IHC assays for Slug also showed that HCC tissues exhibited higher levels of Slug expression; however, normal hepatocellular tissues displayed lower Slug levels (Figure 5C). Slug has been suggested to promote HCC tumorigenesis. According to the TCGA database, correlations between Slug expression and clinical or pathological characteristics exist (Figure 5D‐I).^20^

### 3.7 MALAT1 increases Slug levels via miR‐124‐3p, and miR‐124‐3p is required for MALAT1 to promote metastasis in HCC

**Figure 6:**
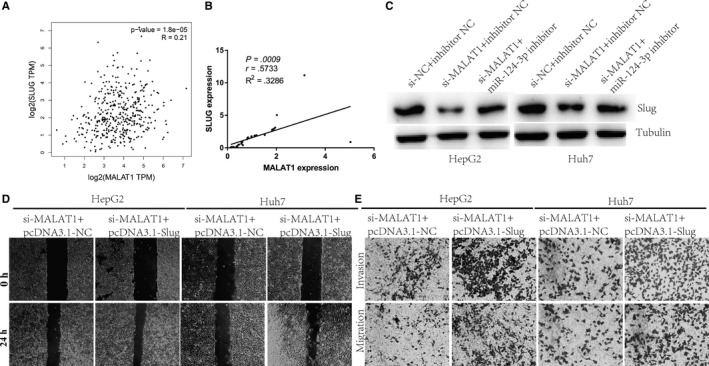
Correlation between MALAT1 and Slug. (A) Pearson's correlation between MALAT1 and Slug in TCGA database. (B) Pearson's correlation between MALAT1 and Slug in HCC and normal tissues. (C) Slug expression was detected by Western blot in HCC cells transfected with si‐NC + inhibitor NC, si‐MALAT1 + inhibitor NC or si‐MALAT1 + miR‐124‐3p inhibitor. Wound healing (D) and transwell assays (E) were performed with HCC cells transfected with si‐MALAT1 + pcDNA3.1‐NC or si‐MALAT1 + pcDNA3.1‐Slug. HCC, hepatocellular carcinoma; MALAT1, metastasis‐associated lung adenocarcinoma transcript 1; NC, negative control

To investigate whether Slug correlated with MALAT1 in HCC cells, we performed an expression analysis. As expected, a positive correlation was found between MALAT1 and Slug expression in HCC using the TCGA database (Figure 6A).^16^ We obtained similar results for 30 HCC tissues (Figure 6B). Similarly, MALAT1 knockdown caused the downregulation of Slug expression, and this dynamics was reversed by transfection with a miR‐124‐3p inhibitor (Figure 6C). This result further confirmed that MALAT1 positively regulates Slug expression via miR‐124‐3p. Further results indicated that increased Slug levels reversed the invasion and metastasis attenuation in HCC cells induced by MALAT1 knockdown (Figure 6D,E) and confirmed that MALAT1 promoted HCC metastasis dependent on Slug.

### 3.8 Inhibition of MALAT1 suppresses Slug expression and HepG2 cell growth in vivo

**Figure 7:**
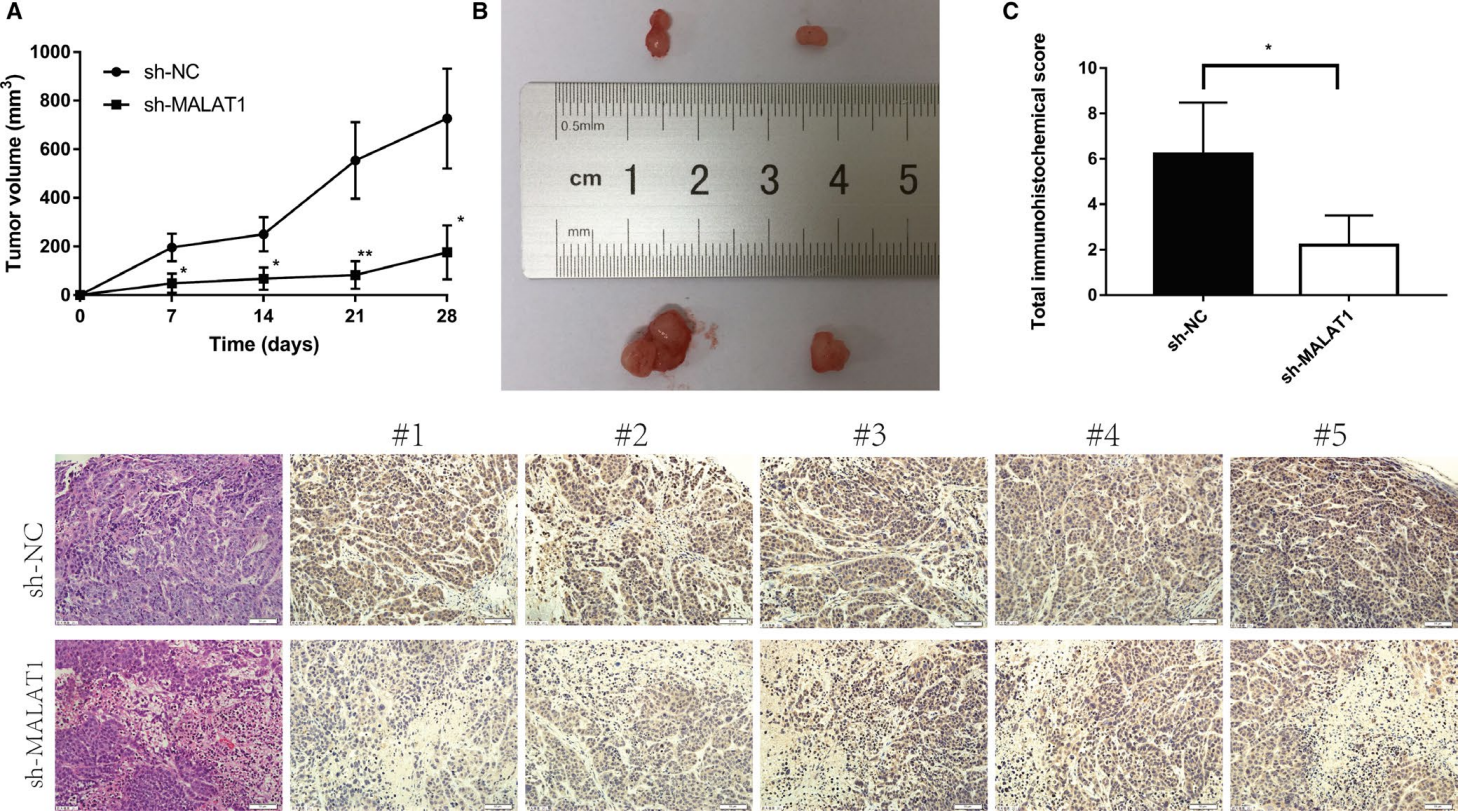
Knockdown of MALAT1 expression inhibits HepG2 cell growth in vivo. (A) Volumes of the HepG2 mouse xenografts of cells transfected with sh‐MALAT1 or sh‐NC were measured every 7 d after injection (*P < .05; **P < .01). (B) Representative images of tumor formation in nude mice bearing HepG2‐shRNAMALAT1 and HepG2‐control cells subcutaneously. (C) H&E analysis shows the tumor section features (20×) and Slug expression in the tumor sections (immunohistochemistry, 20×). *P < .05 vs. sh‐NC. MALAT1, metastasis‐associated lung adenocarcinoma transcript 1; NC, negative control

After transfecting HepG2 cells with sh‐NC or sh‐MALAT1, they were injected subcutaneously into nude mice. The tumor volumes and sizes were greater in the sh‐NC group than in the sh‐MALAT1 group (Figure 7A,B). IHC analysis of nude mouse xenografts showed decreased Slug expression in the sh‐MALAT1 group (Figure 7C). These data indicate that MALAT1 promotes HCC progression by increasing Slug expression in vivo.

## 4 DISCUSSION

Long‐chain noncoding RNA MALAT1 was first identified in the primary non‐small cell lung cancer cells of patients by subtractive hybridization and is highly conserved in mammalian evolution.^21^ Multiple studies have confirmed that the lncRNA MALAT1 is abnormally expressed in many human malignancies and changes the biological phenotypes of tumor cells.^22^, ^23^, ^24^ MALAT1 has varying effects on tumor proliferation, apoptosis, invasion and metastasis, and on drug resistance.^25^ Our data indicate that MALAT1 levels were considerably increased in HCC tumors. High MALAT1 levels were negatively correlated with DFS. MALAT1 knockdown in HCC cells reduced cellular invasion and metastasis. The above results indicate that lncRNA MALAT1 acts as an oncogene in HCC.

Slug (SNAI2) is a transcriptional repressor and a member of the Snail family (transcription factor of C2H2‐type zinc finger). Its biological role is to bind to the E‐box motif of CDH1 to inhibit CDH1 transcription in breast cancer.^15^ This protein was confirmed to be involved in the EMT process to promote tumor metastasis.^26^, ^27^ CDH1 is the main epithelial marker in EMT. High expression of Slug reduces the level of CDH1, promotes the transformation of epithelial cells into mesenchymal cells, and promotes the metastasis of tumor cells. Our data show that Slug was highly expressed in HCC, and Slug expression levels were positively correlated with MALAT1 expression. Decreasing MALAT1 expression decreases Slug levels.

Recently, lncRNAs were reported to regulate gene expression through competitive elements that bind miRNAs. That is, lncRNAs act as endogenous sponges to inhibit the binding between miRNAs and their target genes. Recent studies have shown that the progression of osteosarcoma (miR‐509), glioma (miR‐101), and EMT (miR‐145) is associated with MALAT1 acting as a ceRNA.^28^, ^29^, ^30^ Therefore, we doubted that MALAT1 was also a sponge for miRNAs in HCC. To confirm this, the site of miRNA binding to MALAT1 was determined by bioinformatic analysis, and we selected miR‐124‐3p as the research subject; MALAT1 knockdown increased miR‐124‐3p levels. In contrast, transfection of a mimic of miR‐124‐3p decreased MALAT1 expression levels, indicating that MALAT1 was negatively regulated by miR‐124‐3p. Some studies suggest that miR‐124‐3p is abnormally expressed in many malignancies.^16^, ^31^, ^32^, ^33^ In addition, miR‐124‐3p plays different regulatory functions in different tumor cell types.^34^, ^35^, ^36^ This study confirmed that miR‐124‐3p overexpression could reduce liver cancer cell metastasis and reduce Slug expression. Further experiments suggested that miR‐124‐3p bind to Slug via complementary bases, as well as to the lncRNA MALAT1 sequence. This means that there is a ceRNA relationship among MALAT1, Slug and miR‐124‐3p. MALAT1 regulates the expression level of Slug through miR‐124‐3p, and inhibiting miR‐124‐3p expression reverses the effects of si‐MALAT1 on HCC cells.

The observed positive correlation between MALAT1 and Slug not only enriches our understanding of their functions in tumor development but also provides a more complete understanding of the MALAT1 lncRNA.

In summary, our study provides a promising evidence that MALAT1 can be used to predict poor clinical features and prognosis in patients with liver cancer. MALAT1 increases HCC cell invasion and migration in vivo and in vitro. miR‐124‐3p was confirmed as both a target molecule and a function mediator of MALAT1 in HCC cells. miR‐124‐3p reduced HCC cell metastasis by targeting Slug. Inhibiting miR‐124‐3p reversed the biological function of knocking down MALAT1 in HCC cells. In short, the MALAT1/miR‐124‐3p/Slug axis promoted the cellular processes of HCC cells. The findings in this paper will further enhance the understanding of the mechanism of action of MALAT1 involved in malignant tumor progression and provide new targets for molecular diagnostics and treatment of HCC.

## CONFLICT OF INTEREST

None.

## Data Availability

All data included in this study are available upon request by contact with the corresponding author.
